# Supplementary material for: Impact of individual and environmental factors on dietary or lifestyle interventions to prevent type 2 diabetes development: a systematic review
Source: Commun Med (Lond). 2023 Oct 5;3:133. doi: 10.1038/s43856-023-00363-0 (PMC10551013; doi:10.1038/s43856-023-00363-0)
Supplement: Supplementary file 3 — Description of Additional Supplementary Files [file 43856_2023_363_MOESM3_ESM.docx]

**Impact of individual and environmental factors on dietary or lifestyle interventions to prevent type 2 diabetes development: a systematic review.**

**Title for Supplementary Data 1:** Statistical results extracted for each effect modifier

**Legend for Supplementary Data 1:** Relevant statistical results for evaluating the effect modification of sociodemographic, clinical, behavioural and molecular factors in response to T2D prevention strategies were extracted from all 81 studies. Data was then arranged according to each effect modifier and presented.

**Title for Supplementary Data 2:** Potential modifiers of intervention effect on incidence of T2D (Source data for Figure 2)

**Legend for Supplementary Data 2:** Number of trial participants in the studies which have evaluated the potential effect modifiers of lifestyle, dietary, and supplement interventions on the incidence of type 2 diabetes.
